# Supplementary material for: Cryo-EM structure of the calcium release-activated calcium channel Orai in an open conformation
Source: eLife. 2020 Nov 30;9:e62772. doi: 10.7554/eLife.62772 (PMC7723414; doi:10.7554/eLife.62772)
Supplement: Figure 2—source data 1. [file elife-62772-fig2-data1.docx]

| **Data collection** |  |
| --- | --- |
| Microscope | FEI Titan Krios (at MSKCC) |
| Camera | Gatan K2 Summit |
| Magnification | 22,500 x |
| Voltage (kV) | 300 |
| Electron exposure (e–/Å2) | 76 |
| Defocus range (μm) | -0.9 to -2.5 |
| Pixel size (Å) | 1.0884 |
| Software | Cryosparc 2, RELION 3.0 |
| Symmetry imposed | C3 |
| Initial particle images (no.) | 1,851,514 |
| Particles with 3 Fabs (no.) | 236,132 |
| Final particle images (no.) | 85,614 |
| Map resolution (Å) | 3.3 |
| FSC threshold 0.143 |  |
| **Refinement** |  |
| Software | Phenix 1.18 real-space-refine |
| Initial model used | PDB: 4HKR |
| Map sharpening *B* factor (Å2) | -100.5 |
| Model resolution (Å) | 3.6 |
| FSC threshold | 0.5 |
| Model composition |  |
| Non-hydrogen atoms | 10,534 |
| Protein residues | 1,437 |
| Ligands | 1 |
| Mean *B* factor (Å2) |  |
| Orai | 118.3 |
| Fab | 79.3 |
| R.m.s. deviations |  |
| Bond length (Å) | 0.004 |
| Bond angles (°) | 0.559 |
| **Validation** |  |
| Clashscore | 11 |
| Rotamer outliers (%) | 0 |
| Ramachandran plot |  |
| Favored (%) | 94.6 |
| Allowed (%) | 5.4 |
| Outliers (%) | 0 |
